# Tissue distribution and kinetics of endogenous porphyrins synthesized after topical application of ALA in different vehicles

**DOI:** 10.1038/sj.bjc.6690644

**Published:** 1999-09

**Authors:** A Casas, H Fukuda, A M del C Batlle

**Affiliations:** Centro de Investigaciones sobre Porfirinas y Porfirias (CIPYP) FCEyN (University of Buenos Aires) and CONICET; Ciudad Universitaria, Pabellón II, 2do piso; (1428), Capital Federal, Argentina

**Keywords:** 5-aminolaevulinic acid, ALA-PDT, ALA lotion, ALA cream, topical application

## Abstract

The use of 5-aminolaevulinic acid (ALA) is gaining increasing attention for photosensitization in photodynamic therapy of superficially localized tumours. The aim of this work was to determine the kinetics of porphyrin generation in tissues after topical application of ALA delivered in different vehicles on the skin overlying the tumour and normal skin of mice. Maximal accumulation was found in tumour 3 h after ALA application in both cream and lotion preparations. Normal and overlying tumour skin tissues showed different kinetic patterns, reflecting histological changes when the latter is invaded by tumour cells. Liver, kidney, spleen and blood porphyrins also raised from basal levels, showing that ALA and/or ALA-induced porphyrins reach all tissues after topical application. During the first 24 h of ALA topical application, precursors and porphyrins are excreted by both urine and faeces. ALA lotion applied on the skin overlying the tumour induced higher accumulation of tumoural porphyrins than cream, and lotion applied on normal skin appeared to be the most efficient upon inducing total body porphyrins. This work has demonstrated the great influence of the formulation of ALA vehicle on penetration through the skin. Knowledge of the kinetics of porphyrin generation after different conditions of ALA application is needed for the optimization of diagnosis and phototherapy in human tumours. © 1999 Cancer Research Campaign


*British Journal of Cancer* (1999) **81**(1), 13–18
© 1999 Cancer Research Campaign
Article no. bjoc.1999.0644

### Tissue distribution and kinetics of endogenous porphyrins synthesized after topical application of ALA in different vehicles

**A Casas, H Fukuda and AM del C Batlle**

Centro de Investigaciones sobre Porfirinas y Porfirias (CIPYP) FCEyN (University of Buenos Aires) and CONICET; Ciudad Universitaria, Pabellón II, 2do piso; (1428) Capital Federal, Argentina

**Summary** The use of 5-aminolaevulinic acid (ALA) is gaining increasing attention for photosensitization in photodynamic therapy of superficially localized tumours. The aim of this work was to determine the kinetics of porphyrin generation in tissues after topical application of ALA delivered in different vehicles on the skin overlying the tumour and normal skin of mice. Maximal accumulation was found in tumour 3 h after ALA application in both cream and lotion preparations. Normal and overlying tumour skin tissues showed different kinetic patterns, reflecting histological changes when the latter is invaded by tumour cells. Liver, kidney, spleen and blood porphyrins also raised from basal levels, showing that ALA and/or ALA-induced porphyrins reach all tissues after topical application. During the first 24 h of ALA topical application, precursors and porphyrins are excreted by both urine and faeces. ALA lotion applied on the skin overlying the tumour induced higher accumulation of tumoural porphyrins than cream, and lotion applied on normal skin appeared to be the most efficient upon inducing total body porphyrins. This work has demonstrated the great influence of the formulation of ALA vehicle on penetration through the skin. Knowledge of the kinetics of porphyrin generation after different conditions of ALA application is needed for the optimization of diagnosis and phototherapy in human tumours.

**Keywords:** 5-aminolaevulinic acid; ALA-PDT; ALA lotion, ALA cream; topical application

Photodynamic therapy (PDT) is a promising new treatment modality for cancer using photosensitizers and light irradiation (Dougherty et al, 1975). In addition, the fluorescence of photosensitizing chromophores has been proposed to be used in the visualization of early-stage cancers (Kriegmair et al, 1996).

The endogenous conversion of 5-aminolaevulinic acid (ALA) to protoporphyrin IX (PpIX) has broadened the use of PDT. ALA is frequently applied topically or systemically in PDT of skin tumours (Kennedy et al, 1990; Grant et al, 1993; Wolf et al, 1993). Kennedy et al (1990) have found that ALA in aqueous solution passed readily through abnormal human skin, but not through normal keratin.

In the cystosol two molecules of ALA are combined to form porphobilinogen (PBG) and four molecules of PBG are then bound to form uroporphyrinogen. Next, uroporphyrinogen is converted to coproporphyrinogen, which is taken up by mitochondria to form PpIX. ALA-induced PpIX accumulation has been shown to be preferentially greater in certain tumoural cells (Batlle et al, 1975; Navone et al, 1988; Fukuda et al, 1989) primarily due to the reduced activity of ferrochelatase, the enzyme responsible for the conversion of PpIX into haem (Dailey and Smith, 1984; Van Hillesberg et al, 1992) and a relative enhancement of deaminase activity (Schoenfeld et al, 1988; Navone et al, 1991), which constitutes the biological rationale for the clinical use of the so called ALA-based PDT (ALA-PDT).

PpIX is thought to be the predominant PDT-relevant metabolite formed from the applied ALA (Schoenfeld et al, 1994), although other porphyrins may be potent photosensitizers too (Menon et al, 1990; Fukuda et al, 1992).

Little is known concerning the kinetics of tissue uptake and localization of the porphyrins induced by ALA in vivo after topical application of the precursor on the tumour, neither is there any report on porphyrin and precursor excretion after topical ALA in mice. The aim of this work was to determine the kinetics of porphyrin generation in tissues after topical application of ALA delivered in different vehicles on the tumour and normal skin of mice.

### MATERIALS AND METHODS

### Chemicals

ALA was purchased from Sigma Chemical Co., St Louis, MO, USA. All other chemicals were of analytical grade.

### Animals

Male BALB/c mice 12 weeks old, weighing 20–25 g were used. They were provided with food (Purina 3, Molinos Río de la Plata, Argentina) and water ad libitum. A mammary adenocarcinoma (Scolnik et al, 1984) (M2, Hospital Roffo, Buenos Aires,

*Received 29 January 1999*
*Revised 22 February 1999*
*Accepted 2 March 1999*

*Correspondence to:* AM Batlle, Viamonte 1881 10A, 1056 Buenos Aires, Argentina

Argentina) was propagated by serial transplantation into male BALB/c mice. Experiments were performed at day 12 after implantation. Tumours of the same uniform size were employed. Animals received human care and were treated in accordance with guidelines established by the Animal Care and Use Committee of the Argentine Association of Specialists in Laboratory Animals (AADEALC), in full accord with the UK Guidelines for the Welfare of Animals in Experimental Neoplasia (UKCCCR, 1988).

### Preparation of ALA

The hydrochloric acid (HCl) salt of ALA was dissolved in sterile saline at a concentration of 100 mg $ml^{-1}$ (lotion) or in a 20% formulation in a base cream (Genargen, Argentina) just prior to application. The pH of both ALA cream and lotion was 3.5.

### ALA administration

For pharmacokinetics of ALA-induced porphyrins, mice received either 10 mg ALA (50 mg of 20% ALA cream) or 15 mg (0.15 ml of 100 mg ALA per ml lotion). Both ALA formulations were applied either on the skin overlying the tumour or on an area of 2.2 $cm^2$ of normal skin, having shaved the hair, rubbing with a smooth paintbrush for a period of 180 s. At different times up to 24 h after ALA administration (three mice for each point) the animals were killed by cervical dislocation.

For dose–response kinetic experiments, 50 mg of a cream containing 10, 20, 30 or 40% ALA or 0.1 ml of a 50, 100, 150 or 200 mg ALA per ml lotion were applied either on the skin overlying the tumour or on the normal skin, contralateral to the tumour, delivering a total amount of 5, 10, 15 and 20 mg ALA. Mice were killed 3 h after ALA administration (three mice for each point). Blood was extracted and tumour, skin (contralateral of the tumour side), skin overlying the tumour, liver, kidney and spleen were removed and maintained at 0°C until extraction of porphyrins.

### Tissue extraction of porphyrins

Before killing, mice were injected with heparin (0.15 ml, 100 UI) and after sacrifice, they were perfused with 200 ml of sterile saline. The tissue samples were homogenized in a 4:1 solution of ethyl acetate–glacial acetic acid mixture (1:5 or 1:10 w/v). Blood samples were heparinized and vigorously vortexed with the same extraction solvents. The mixtures were centrifuged for 30 min at 3000 ***g***, and the supernatants were added with an equal volume of HCl 5%. Extraction with HCl was repeated until there was no detectable fluorescence in the organic layer. The aqueous fraction was used for the spectrophotometric determination of porphyrins. The sum of absorptions at 380 and 430 nm was substracted from twice the Soret band maximum, and the difference, divided by a correction factor for Uroporphyrin (Falk, 1964). A standard of Uroporphyrin I was used as reference.

### Porphyrins and precursors determinations in urine

The concentration of total porphyrins and precursors in urine were determined by column elution and subsequent spectrophotometrical measurement according to the methods of Doss and Schmidt (1971*a*, 1971*b*).

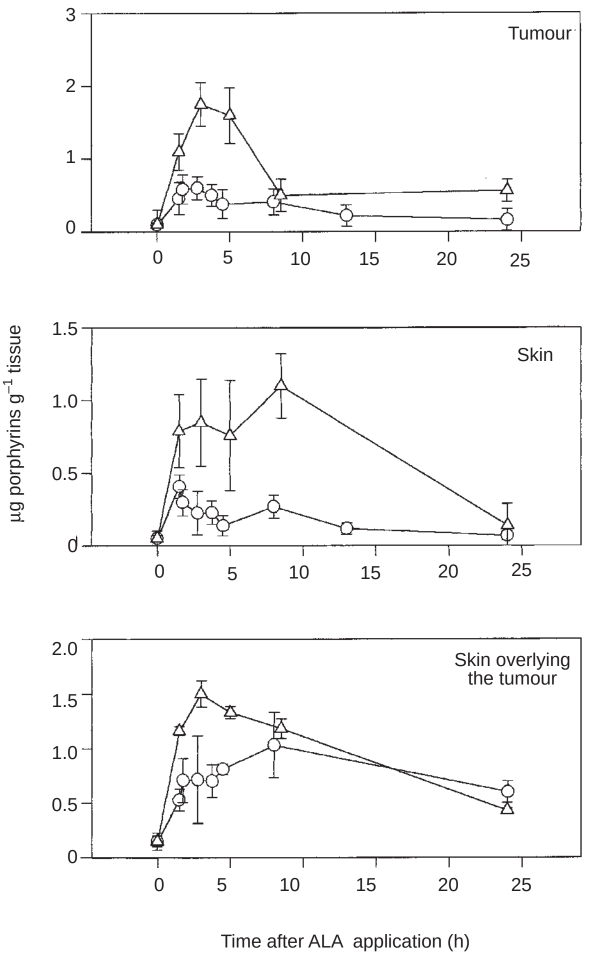

**Figure 1** Concentration of ALA-induced porphyrins in tumour, normal skin and skin overlying the tumour as a function of time after topical application of ALA in cream and lotion on the skin overlying the tumour. At different times after topical application of 10 mg ALA in cream (○) or 15 mg in lotion (Δ) on the skin overlying the tumour, the tissues were excised and porphyrins extracted as detailed in Materials and Methods. Each data point represents the average of three determinations. Error bars show standard deviations

### Porphyrin determination in faeces

The content of total porphyrins in faeces was determined spectrophotometrically according to Batlle and Grinstein (1962) after extraction with chloroform of the esterified porphyrins (With, 1975).

### Statistical analysis

The unpaired *t*-test was used to established the significance of differences between groups. Differences were considered statistically significant when $P < 0.05$.

### RESULTS

The time course of porphyrin levels in tissues, after topical application of ALA on skin overlying the tumour are shown in Figures 1, 2 and 3. Maximal accumulation was found in tumour 3 h after ALA application in both cream and lotion (Figure 1). Normal and overlying tumour skin tissues showed different kinetic patterns.

© 1999 Cancer Research Campaign

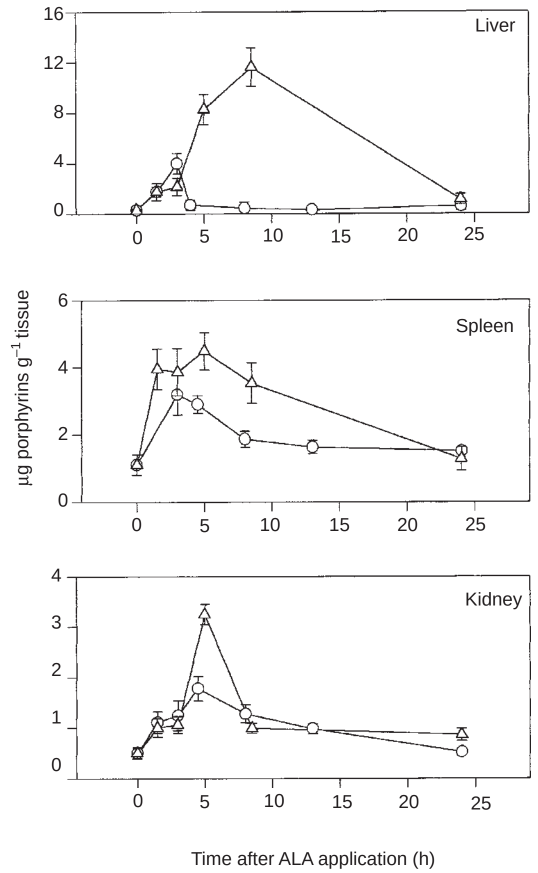

**Figure 2** Concentration of ALA-induced porphyrins in liver, spleen and kidney as a function of time after topical application of ALA in cream and lotion on skin overlying the tumour. At different times after topical application of 10 mg ALA in cream (○) or 15 mg in lotion (Δ) on the skin overlying the tumour, the tissues were excised and porphyrins extracted as detailed in Materials and Methods. Each data point represents the average of three determinations. Error bars show standard deviations.

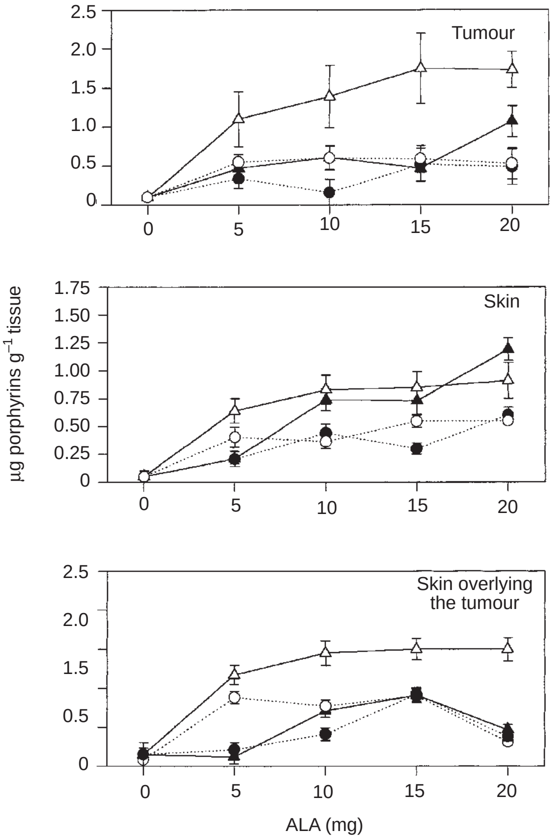

**Figure 4** Porphyrin accumulation in tumour, skin and skin overlying the tumour after topical application on the skin overlying the tumour and normal skin of various quantities of ALA. Different amounts of ALA cream were applied on the skin overlying the tumour (○), or normal skin (●) and of ALA lotion on the skin overlying the tumour (Δ) and normal skin (▲). Three hours later, tissues were excised and porphyrins extracted as detailed in Materials and Methods. Each data point represents the average of three determinations. Error bars show standard deviations.

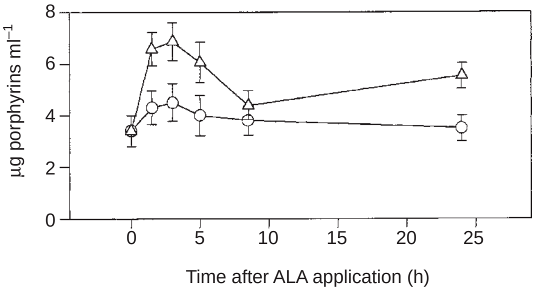

**Figure 3** Concentration of ALA-induced porphyrins in blood as a function of time after topical application of ALA in cream and lotion on the skin overlying the tumour. At different times after tumour topical application of 10 mg ALA in cream (○) or 15 mg in lotion (Δ) on the skin overlying the tumour, heparinized blood was extracted for porphyrin determinations as detailed in Materials and Methods. Each data point represents the average of three determinations. Error bars show standard deviations

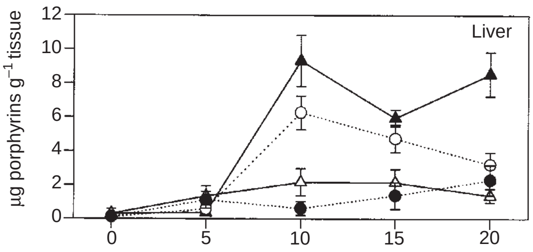

**Figure 5** Porphyrin accumulation in liver after topical application on the skin overlying the tumour and normal skin of various quantities of ALA. Different amounts of ALA cream were applied on the skin overlying the tumour (○), or normal skin (●) and of ALA lotion on the skin overlying the tumour (Δ) and normal skin (▲). Three hours later, livers were excised and porphyrins extracted as detailed in Materials and Methods. Each data point represents the average of three determinations. Error bars show standard deviations

While ALA cream-induced porphyrins peaked in normal skin at 1.5 h after application, ALA lotion produced a broad peak between 3 and 8 h. Conversely, peak levels in skin overlying the tumour were reached at 3 and 8 h after ALA lotion and cream application respectively.

Figure 2 depicts kinetic profiles in liver, spleen and kidney. Liver exhibits a maximum accumulation of tetrapyrroles at 3 h and 8 h after cream and lotion application respectively, spleen and kidney show nearly coincident peaks at about 5 h for both vehicles of ALA.

*© 1999 Cancer Research Campaign*

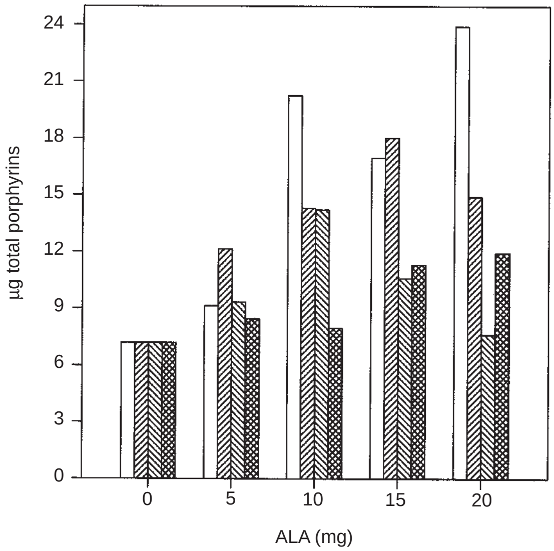

**Figure 6** Total porphyrin accumulation after topical application in the skin overlying the tumour and normal skin of different amounts of ALA. Three hours after application of varying amounts of ALA cream on the skin overlying the tumour (▨), or normal skin (▩) and of ALA lotion on the skin overlying the tumour (▧) and normal skin (□), the sum of porphyrin concentrations in skin, tumour, skin overlying the tumour, kidney, liver, spleen and blood were depicted

**Table 1** Porphyrin excretion after topical application of ALA on the skin overlying the tumour

| | Urine | Faeces |
|---|---|---|
| Control | 0.084 ± 0.009 | 35.34 ± 5.50 |
| Topical ALA | 1.82 ± 0.12 | 93.88 ± 7.32 |

Total amount of porphyrins in µg excreted in 24 h per mice after application of 15 mg of ALA lotion on the skin overlying the tumour.

**Table 2** Precursors excretion after topical application of ALA on the skin overlying the tumour

| | ALA | PBG |
|---|---|---|
| Control | ND | ND |
| Topical ALA | 3.61 ± 0.45 | 39.48 ± 8.04 |

Total amount of ALA and PBG in µg excreted in 24 h per mice after application of 15 mg of ALA lotion on the skin overlying the tumour.
ND: non-detectable.

Porphyrin levels always return to normal values within 24 h of ALA topical application whichever vehicle was employed.

Blood porphyrin values (Figure 3) found its maximum at 3 h and then fell to basal levels when cream vehicle was employed. A slight increase was observed at 24 h after ALA lotion application.

The dose course of porphyrin levels in tissues after cream and lotion application of ALA on either skin overlying the tumour or normal skin are shown in Figures 4, 5 and 6. In tumour, skin and skin overlying the tumour (Figure 4), maximum levels were higher when ALA was applied in lotion on skin overlying the tumour. Porphyrin formation reached a plateau in tumour between 10 mg and 15 mg of ALA when either ALA lotion or cream were applied. Tumoural porphyrins induced when ALA was applied on normal skin peaked at 20 mg and 10 mg for lotion and cream respectively.

In skin, ALA lotion induced higher accumulation than ALA cream. In skin overlying the tumour maximal values were obtained when ALA lotion was applied on it.

Figure 5 shows that maximal liver porphyrin accumulation was obtained after topical application of ALA lotion on the normal skin. High amounts of tetrapyrroles were also formed after application of ALA cream on skin overlying the tumour, in both cases yielding a saturation point at 10 mg ALA.

Figure 6 represents the total amount of porphyrins accumulated in all tissues analysed. Both cream and lotion applied on normal skin induced increasing levels of tissue porphyrins while increasing the dose of ALA, but when applied on the skin overlying the tumour, the values decreased with high ALA doses, reflecting a saturation pattern. Higher total amounts of porphyrins were formed after application of ALA lotion on skin overlying the tumour and in normal skin.

Table 1 shows total excretion of porphyrins during the first 24 h after ALA lotion application on skin overlying the tumour, an increase from 0.084 to 1.82 µg in urine and from 35.34 to 93.88 µg in faeces of ALA-treated animals compared to the controls was found; 24 to 48 h after ALA application values returned nearly to basal levels.

Table 2 shows total excretion of precursors during the first 24 h after ALA application. Whereas precursor levels are undetectable in control mice, after topical application of ALA lotion values raise to 3.61 µg for ALA and 39.48 µg for PBG; 24–48 h after ALA application, precursor levels were again undetectable.

### DISCUSSION

Topical application of ALA lotion on skin overlying the tumour, induced peaks of porphyrin accumulation in tumour and skin overlying the tumour 3 h after treatment, in agreement with Peng et al (1992) and Henderson et al (1995), who have found that ALA-induced porphyrins in murine adenocarcinomas peaked between 3 and 5 h post-application. We have found that administration of 15 mg ALA lotion, formed nearly 2 µg porphyrins $g^{-1}$ tissue, twice the amount reported by Peng et al (1992) using a 20% ALA cream in a mammary adenocarcinoma. These porphyrins may then reach spleen and kidney at 5 h, as can be deduced from its delayed peak in these tissues. Peak porphyrin levels in liver and skin were found even later (8 h). Apparently, high amounts of porphyrins are synthesized in the liver, which can then be redistributed and accumulated in skin tissue.

In line with these results, Fukuda et al (1992) and Peng et al (1992) reported that after intraperitoneal ALA administration, peak porphyrin levels are found in tumour at shorter intervals than in liver, indicating that tumoural cells are capable of synthesizing the endogenous porphyrins themselves.

Spleen and kidney did not show a second peak after 8 h, showing that liver porphyrins had already been excreted and did not accumulate in these tissues. A rise in blood porphyrin levels at 24 h supports the hypothesis that liver porphyrins are being eliminated.

A dynamic flow of porphyrins is highly probable, given the serum porphyrin-binding molecules and the ease at which ALA-induced porphyrins can exit from tissue (Fukuda et al, 1993; Henderson et al, 1995). However, it cannot be ruled out the possibility that ALA itself has reservoir compartments such as the

 © 1999 Cancer Research Campaign stratum corneum in skin (Rougier and Lotte, 1986) which may lead to particular kinetic patterns in each tissue.

We have found that high quantities of porphyrins were accumulated in a tumour implanted in the contralateral side of a topically treated tumour (data not shown), indicating that, if ALA is not transferred to remote sites by direct contact, an efflux of porphyrins to distant tumours may exist, very likely through the bloodstream. Similar results were obtained by Stringer et al (1996), when psoriasis plaques were treated with ALA and remote plaques displayed significant fluorescence, afterwards.

Topical application of ALA cream generated lower amounts of porphyrins than ALA lotion in all tissues. In this case both tumour and liver porphyrins peak at 3 h, while delayed peaks were observed in skin overlying the tumour and kidney and no rise was found in blood porphyrin levels at 24 h. Probably, the amount of ALA in this vehicle entering into the bloodstream is lower, thus accounting for the lack of extensive synthesis of liver porphyrins.

Porphyrin synthesis is most efficient in tumour when ALA lotion is applied. Moreover, it is noteworthy that ALA lotion applied on normal skin distant from tumour, induced levels of tumoural porphyrins equal or higher than those induced by ALA cream directly applied on the skin overlying the tumour. We hypothesize that ALA penetration is favoured by a hydrophilic vehicle and that after passing through the stratum corneum and adnexal structures, reaches the circulation and it is distributed homogeneously by the tumour microvasculature, so producing higher porphyrin levels than a lipophilic vehicle.

Similarly, normal skin accumulates higher amount of porphyrins after application of an ALA lotion on both skin overlying the tumour and normal skin, surpassing levels reached with the cream application directly over the skin, indicating the important role of the vehicle. Skin overlying the tumour always showed similar kinetic patterns as to the tumour, reflecting the histological changes occurring in this tissue when it is invaded by tumour cells.

In all tissues analysed ALA lotion on normal skin produced saturation patterns with high quantities of ALA (20 mg) as compared to its application on skin overlying the tumour, probably because of the larger retention of porphyrins in tumour, allowing a lesser amount of ALA to be distributed among the rest of the tissues.

The sum of porphyrins accumulated in all tissues shows that maximal porphyrin values are formed when ALA is applied in the lotion formulation. This assures a better penetration and distribution after passing through both normal and overlying tumour skins. Indeed, the fact that maximal values are reached after application on normal skin indicates that ALA would be penetrating easier normal skin than invaded overlying tumour skin.

Results indicate that in control mice, porphyrins are mostly eliminated by faeces (35.34 μg against 0.084 μg measured in urine), but in ALA-topically treated animals, control values increase three times in faeces and 21 times in urine, showing that hydrophilic porphyrins (uroporphyrins) are formed and more rapidly eliminated in this way. From the precursor excretion profiles we can also speculate that PBG deaminase and not ALA dehydrase is the limiting enzyme in the conversion of ALA to porphyrins.

Intratumour and i.p. administration of ALA in the same adenocarcinoma gave different kinetic biodistribution profiles and total amounts of ALA-derived porphyrins in tissues (Fukuda et al, 1992), showing that the route of ALA administration strongly affects tetrapyrrole synthesis, distribution and elimination patterns.

In contrast to Kennedy et al (1990) findings about the relative impermeability of the normal human skin to ALA and Fritsch et al (1996) who reported lack of increase of either porphyrins or precursors in urine and blood of topically treated patients, we have found that ALA is capable of penetrating normal mouse skin, to enter the bloodstream and reach the tissues. Moreover, it is thought that keratin, due to its fibrilar structure, may exist in two phases: one polar and another apolar; the former enriched in water is actually contained into the latter. This may account for penetration of hydrophilic substances such as ALA into the stratum corneum (Torralba Rodríguez, 1985).

The small size of the animal used, the thin epidermis, the higher density of hair follicles and the relative large body surface covered by the ALA formulation may explain the differences observed between human and mice response. These differences must be taken into account when extrapolating time and dose schedules to human studies. Moreover, BALB/c albino mice skin may be less resistant to penetration of ALA, in the same way that the more fair and thin human skins allow quite significant penetration of ALA (Kennedy and Pottier, 1992). This may also explain the results of Goff et al (1992), who found no evidence of PpIX fluorescence in hairless guinea pig skin exposed to topical ALA.

Penetration of ALA appears to be greatly influenced by the vehicle, and may be the result of penetration of ALA through the stratum corneum either by diffusion or active transport or direct passage to the dermis via adnexal structures, as it was reported for polar penetrants (Magee, 1991).

We have presented here strong evidence for the great influence of the formulation of ALA vehicle on its penetration through the skin. Knowledge of the kinetics of porphyrin generation after different conditions of ALA application is important for the optimization of diagnosis and phototherapy of human tumours by means of ALA-PDT.

### ACKNOWLEDGEMENTS

This research was supported by grants from the Argentine National Research Council (CONICET), the Association for International Cancer Research (AICR)-UK and the Alberto J Roemmers Foundation, Argentine. The authors are very grateful to Mrs Victoria Castillo for her skillful technical assistance. AM del CB and HF hold the posts of Superior and Associate Researchers at the CONICET. AC is a CONICET Research Fellow.

### REFERENCES

Batlle A and Grinstein M (1962) Porphyrin biosynthesis. Studies on the nature of a biosynthetic porphyrin and its identification with the so-called “208” porphyrin. *Biochim Biophys Acta* **57**: 191–194

Batlle A, Llambías E, Wider E and Tigier H (1975) Porphyrin biosynthesis in the soybean callus tissue system XV. The effect of growth conditions. *Int J Biochem* **6**: 591–606

Dailey H and Smith A (1984) Differential interaction of porphyrins used in photoradiation therapy with ferrochelatase. *Biochem J* **223**: 441–445

Doss M and Schmidt A (1971*a*) Rapid determination of urinary total porphyrins by ion exchange chromatography. *Z Klin Chem Klin Biochem* **9**: 415–418

Doss M and Schmidt A (1971*b*) Quantitative Bestimmung der 5-Aminolävulinsäure und Porphobilinogen im Urin mit lonenaustauschercromathographie-Fertigsäulen. *Z Klin Chem Klin Biochem* **9**: 99–102

Dougherty T, Grindey G, Fiel R, Weishaupt K and Boyle D (1975) Photoradiation therapy II: cure of animal tumours with hematoporphyrin and light. *J Natl Cancer Inst* **55**: 115–121

Falk J (1964) Porphyrins and metalloporphyrins. In: *Biochimica of Biophysica Acta-Library* Vol. 2. Elsevier: Amsterdam

Fritsch C, Verwohlt B, Bolsen K, Ruzicka T and Goerz G (1996) Influence of topical photodynamic therapy with 5-aminolevulinic acid on porphyrin metabolism. *Arch Dermatol Res* **288**: 517–521

Fukuda H, Paredes S and Batlle A (1989) Tumour-localizing properties of porphyrins. In vitro studies using the porphyrin precursor, aminolevulinic acid, in free and liposome encapsulated forms. *Drug Des Deliv* **5**: 133–139

Fukuda H, Paredes S and Batlle A (1992) Tumour-localizing properties of porphyrins. In vivo studies using free and liposome encapsulated aminolevulinic acid. *Comp Biochem Physiol* **102B**: 433–436

Fukuda H, Batlle A and Riley P (1993) Kinetics of porphyrin accumulation in cultured epithelial cells exposed to ALA. *Int J Biochem* **25**: 1407–1410

Goof B, Bachor R, Kollias N and Hasan T (1992) Effects of photodynamic therapy with topical application of 5-aminolevulinic acid on normal skin of hairless guinea pigs. *J Photochem Photobiol* **15**: 239–251

Grant W, Hopper C, MacRobert A, Speight P and Bown S (1993) Photodynamic therapy of oral cancer: photosensitization with systemic aminolevulinic acid. *Lancet* **342**: 147–148

Henderson BW, Vaughan L, Bellnier D, Van Leengoed H, Johnson PG and Oseroff AR (1995) Photosensitization of murine tumour, vasculature and skin by 5-aminolevulinic acid-induced porphyrin. *Photochem Photobiol* **62**: 780–789

Kennedy J and Pottier R (1992) Endogenous protoporphyrin IX, a clinically useful photosensitizer for photodynamic therapy. *J Photochem Photobiol B* **14**: 275–292

Kennedy J, Pottier R and Pross G (1990) Photodynamic therapy with endogenous protoporphyrin IX: basic principles and present clinical experience. *J Photochem Photobiol B* **6**: 143–148

Kriegmair M, Baumgartner R, Knuechel R, Stepp H, Hofstädter F and Hofstetter A (1996) Detection of early bladder cancer by 5-aminolevulinic acid induced porphyrin fluorescence *J Urology* **155**: 105–110

Magee P (1991) Percutaneous absorption: critical factors in transdermal transport. In: *Dermatoxicology*, Marzulli F and Maibach H (eds) pp. 1–35. Hemisphere: New York

Menon J, Becker S, Persad S and Haberman H (1990) Quantitation of hydrogen peroxide formed during UV-visible irradiation of protoporphyrin, coproporphyrin and uroporphyrin. *Clin Chim Acta* **186**: 375–381

Navone N, Frisardi Resnik E, Batlle A and Polo C (1988) Porphyrin biosynthesis in human breast cancer. Preliminary mimetic in vitro studies. *Med Sci Res* **16**: 61–62

Navone N, Polo C, Frisardi A and Batlle A (1991) Mouse mammary carcinoma PBGase and hydroxymethylbilane synthetase. *Comp Biochem Physiol B* **98**: 67–71

Peng Q, Moan J, Warloe T, Nesland JM and Rimington C (1992) Distribution and photosensitizing efficiency of porphyrins induced by application of exogenous 5-aminolevulinic acid in mice bearing mammary carcinoma. *Int J Cancer* **52**: 433–443

Rougier A and Lotte C (1986) Correlations between horny layer concentration and percutaneous absorption. In: *Pharmacology and Skin*: *1. Skin Pharmacokinetics.* Shroot B (ed) Schaeger: Switzerland

Schoenfeld N, Epstein O, Lahav M, Mamet R, Shaklai M and Atsmon A (1988) The heme biosynthetic pathway in lymphocytes of patients with malignant lymphoproliferative disorders. *Cancer Lett* **43**: 43–48

Schoenfeld N, Mamet R, Nordenberg Y, Shafran M, Babushkin T and Malik Z (1994) Protoporphyrin biosynthesis in melanoma B16 cells stimulated by 5-aminolaevulinic acid and chemical inducers: characterization of photodynamic inactivation. *Int J Cancer* **56**: 106–112

Scolnik A, Rubio M, Colombo L, Comolli R and Caro R (1984) Further studies on the hystamine metabolism in the M2 adenocarcinoma. *Biomed Pharmacother* **38**: 465–467

Stringer M, Collins P, Robinson D, Stables G and Sheehan-dare R (1996) The accumulation of Protoporphyrin IX in plaque psoriasis after topical application of 5-aminolevulinic acid indicates a potential for superficial photodynamic therapy. *J Invest Dermatol* **107**: 76–81

Torralba Rodríguez A (1985) Histología y fisiología de la piel y sus anejos. Aspectos básicos e implicaciones cosméticas. In: *Cosmetología teórico-práctica*, pp 13–46. Consejo General de Colegios Oficiales de Farmacéuticos: Madrid

UK Coordinating Committee on Cancer Research (1988) *UKCCCR Guidelines for the Welfare of Animals in Experimental Neoplasia.* UKCCCR: London

Van Hillesberg R, Van der Berg J, Kort W, Terpstra O and Wilson J (1992) Selective accumulation of endogenously produced porphyrins in a liver metastasis model in rats. *Gastroenterology* **103**: 647–651

With T (1975) Clinical use of porphyrin ester cromatography in urine and feces. *Dan Med Bull* **22**: 74

Wolf P, Rieger E and Kerl H (1993) Topical photodynamic therapy with endogenous porphyrins after application of 5-aminolevulinic acid. *J Am Acad Dermatol* **28**: 17–21

*© 1999 Cancer Research Campaign*

## References

[PDF_00497] DEL BATLLE A. M., GRINSTEIN M. (1962). Porphyrin biosynthesis. Studies on the nature of a biosynthetic porphyrin and its identification with the so-called "208" porphyrin.. Biochim Biophys Acta.

[PDF_00503] Dailey H. A., Smith A. (1984). Differential interaction of porphyrins used in photoradiation therapy with ferrochelatase.. Biochem J.

[PDF_00505] Doss M., Schmidt A. (1971). Rapid determination of urinary total porphyrins by ion exchange chromatography.. Z Klin Chem Klin Biochem.

[PDF_00510] Dougherty T. J., Grindey G. B., Fiel R., Weishaupt K. R., Boyle D. G. (1975). Photoradiation therapy. II. Cure of animal tumors with hematoporphyrin and light.. J Natl Cancer Inst.

[PDF_00517] Fritsch C., Verwohlt B., Bolsen K., Ruzicka T., Goerz G. (1996). Influence of topical photodynamic therapy with 5-aminolevulinic acid on porphyrin metabolism.. Arch Dermatol Res.

[PDF_00526] Fukuda H., Batlle A. M., Riley P. A. (1993). Kinetics of porphyrin accumulation in cultured epithelial cells exposed to ALA.. Int J Biochem.

[PDF_00520] Fukuda H., Paredes S., Batlle A. M. (1989). Tumor-localizing properties of porphyrins. In vitro studies using the porphyrin precursor, aminolevulinic acid, in free and liposome encapsulated forms.. Drug Des Deliv.

[PDF_00531] Grant W. E., Hopper C., MacRobert A. J., Speight P. M., Bown S. G. (1993). Photodynamic therapy of oral cancer: photosensitisation with systemic aminolaevulinic acid.. Lancet.

[PDF_00534] Henderson B. W., Vaughan L., Bellnier D. A., van Leengoed H., Johnson P. G., Oseroff A. R. (1995). Photosensitization of murine tumor, vasculature and skin by 5-aminolevulinic acid-induced porphyrin.. Photochem Photobiol.

[PDF_00537] Kennedy J. C., Pottier R. H. (1992). Endogenous protoporphyrin IX, a clinically useful photosensitizer for photodynamic therapy.. J Photochem Photobiol B.

[PDF_00540] Kennedy J. C., Pottier R. H., Pross D. C. (1990). Photodynamic therapy with endogenous protoporphyrin IX: basic principles and present clinical experience.. J Photochem Photobiol B.

[PDF_00549] Menon I. A., Becker M. A., Persad S. D., Haberman H. F. (1990). Quantitation of hydrogen peroxide formed during UV-visible irradiation of protoporphyrin, coproporphyrin and uroporphyrin.. Clin Chim Acta.

[PDF_00558] Peng Q., Moan J., Warloe T., Nesland J. M., Rimington C. (1992). Distribution and photosensitizing efficiency of porphyrins induced by application of exogenous 5-aminolevulinic acid in mice bearing mammary carcinoma.. Int J Cancer.

[PDF_00568] Schoenfeld N., Mamet R., Nordenberg Y., Shafran M., Babushkin T., Malik Z. (1994). Protoporphyrin biosynthesis in melanoma B16 cells stimulated by 5-aminolevulinic acid and chemical inducers: characterization of photodynamic inactivation.. Int J Cancer.

[PDF_00572] Scolnick A. J., Rubio M. C., Colombo L. L., Comolli R. R., Caro R. A. (1984). Further studies on the histamine metabolism in the M-2 adenocarcinoma.. Biomed Pharmacother.

[PDF_00584] Van Hillegersberg R., Van den Berg J. W., Kort W. J., Terpstra O. T., Wilson J. H. (1992). Selective accumulation of endogenously produced porphyrins in a liver metastasis model in rats.. Gastroenterology.

[PDF_00587] With T. K. (1975). Clinical use of porphyrin ester cromatography of urine and feces.. Dan Med Bull.

